# 

**Published:** 1899-08-12

**Authors:** 


**CADBURY'S COCOA**
ABSOLUTELY PURE.

### THE HOSPITAL

**CADBURY'S COCOA**
NO DRUGS OR ALKALIES USED.

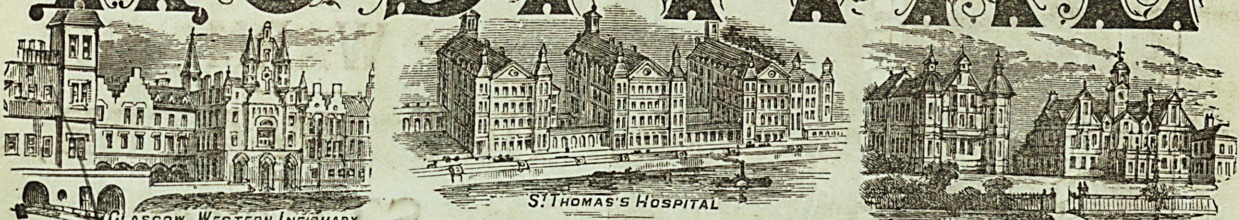

No. 672. Vol. XXVI. [Registered as a Newspaper.] SATURDAY, AUGUST 12TH, 1899. [Subscription Terms, see p. 326.] PRICE TWOPENCE.

